# Digital pathways to healthcare: a systematic review for unveiling the trends and insights in online health information-seeking behavior

**DOI:** 10.3389/fpubh.2025.1497025

**Published:** 2025-02-12

**Authors:** Yang Fu, Ping Han, Jian Wang, Fakhar Shahzad

**Affiliations:** ^1^School of Economics, Harbin University of Commerce, Harbin, China; ^2^School of Accountancy, Harbin Finance University, Harbin, China; ^3^College of Economics and Management, Zhengzhou University of Light Industry, Zhengzhou, China; ^4^Research Institute of Business Analytics and Supply Chain Management, College of Management, Shenzhen University, Shenzhen, China

**Keywords:** health information, CiteSpace, information behavior, online, health information-seeking behavior

## Abstract

The importance of seeking online health information cannot be overstated when addressing public health concerns. Researchers must comprehensively review the literature on online health information seeking to fully comprehend the underlying behaviors and trends that shape this phenomenon. This systematic review utilizes bibliometric methodologies and the scientometric software CiteSpace to thoroughly analyze journals from the Web of Science core collection database (*n* = 2,761), providing the theoretical groundwork for future research in this field. Three main findings emerged from the analysis: first, research on online health information-seeking behavior has steadily increased, indicating that it is a hot topic in academia. Second, the convergence trend with emerging information technologies such as big data, artificial intelligence, and social media is changing user behavior and how people search for health information. Third, there is a growing emphasis on understanding how factors such as the digital divide, social media influence, public health initiatives, risk perception, and health anxiety affect online health information-seeking behavior. The research suggests potential areas for future investigation, such as emerging technologies, digital inequalities, social media analysis, public health implications, and psychological factors in health information-seeking. These areas have the potential to inform evidence-based interventions and advance the understanding of critical issues in healthcare.

## 1 Introduction

In April 2022, the “14th Five-Year Plan” National Health Plan, compiled by the State Council by the “Healthy China 2030” Planning Outline and others, proposed that people’s life safety and physical health should be given top priority (1). National health has become a cornerstone of China’s social development and prosperity. In addition, citizens have gradually begun to attach importance to their health management and actively use information technology for online health information-seeking behavior (OHISB). According to the “2022 China Online Health and Medical Service Consumption White Paper” released by JD Health and Research Institute, as of December 2021, online medical users have reached 298 million, with a year-on-year increase of 38.7%. The emergence of information technologies such as the Internet has changed the traditional mode of medical and health services for users. The OHISB can meet users’ needs for content, diagnosis and treatment, medication, and health management (2).

At present, OHISB has become a research hotspot for many scholars. Still, the review of relevant literature mainly focuses on research in a certain sub-field, such as a meta-analysis of factors affecting users’ OHISB (3), a systematic discussion of the relationship between online health information seeking and the doctor-patient relationship (4), review of research on health literacy and OHISB (5) and systematic analysis of OHISB (6). Due to different focuses, these studies still have limitations in the following two aspects: First, existing studies mainly conduct research from a certain perspective and lack a comprehensive and systematic review of OHISB (3, 7, 30). Second, existing reviews lack the use of scientific measurement software for objective and systematic analyses and summaries (31, 32), particularly in the current study context. Therefore, with this comprehensive approach (which combines bibliometric analysis and the use of CiteSpace software), this study embodies a groundbreaking effort to close the current research gaps on OHISB. The key objective is to answer the following research questions.

RQ1: How does OHISB evolve regarding key trends, emerging topics, and knowledge networks?

RQ2: What are its practical implications for improving health literacy and public health outcomes?

This study used the CiteSpace software to systematically sort out relevant literature on OHISB in the Web of Science (WoS). The clear and rigorous approach presented here enhances the reliability of academic research in the field of OHISB, maintaining credibility and reproducibility requirements. The results provide interdisciplinary insights, identify emerging trends, and map knowledge networks within the subject by combining multiple perspectives from the existing studies. Furthermore, it explains the structural dynamics of OHISB, providing important insights into research pathways and thematic linkages. The research has practical implications for policymakers, healthcare practitioners, and information workers beyond academic discourse. These impacts encourage the development of measures to improve health literacy and inform evidence-based decision-making. Finally, this study significantly improves public health outcomes through multidisciplinary collaboration, information exchange, and a deeper understanding of this complex phenomenon.

## 2 Research methods and data sources

**Figure 1:**
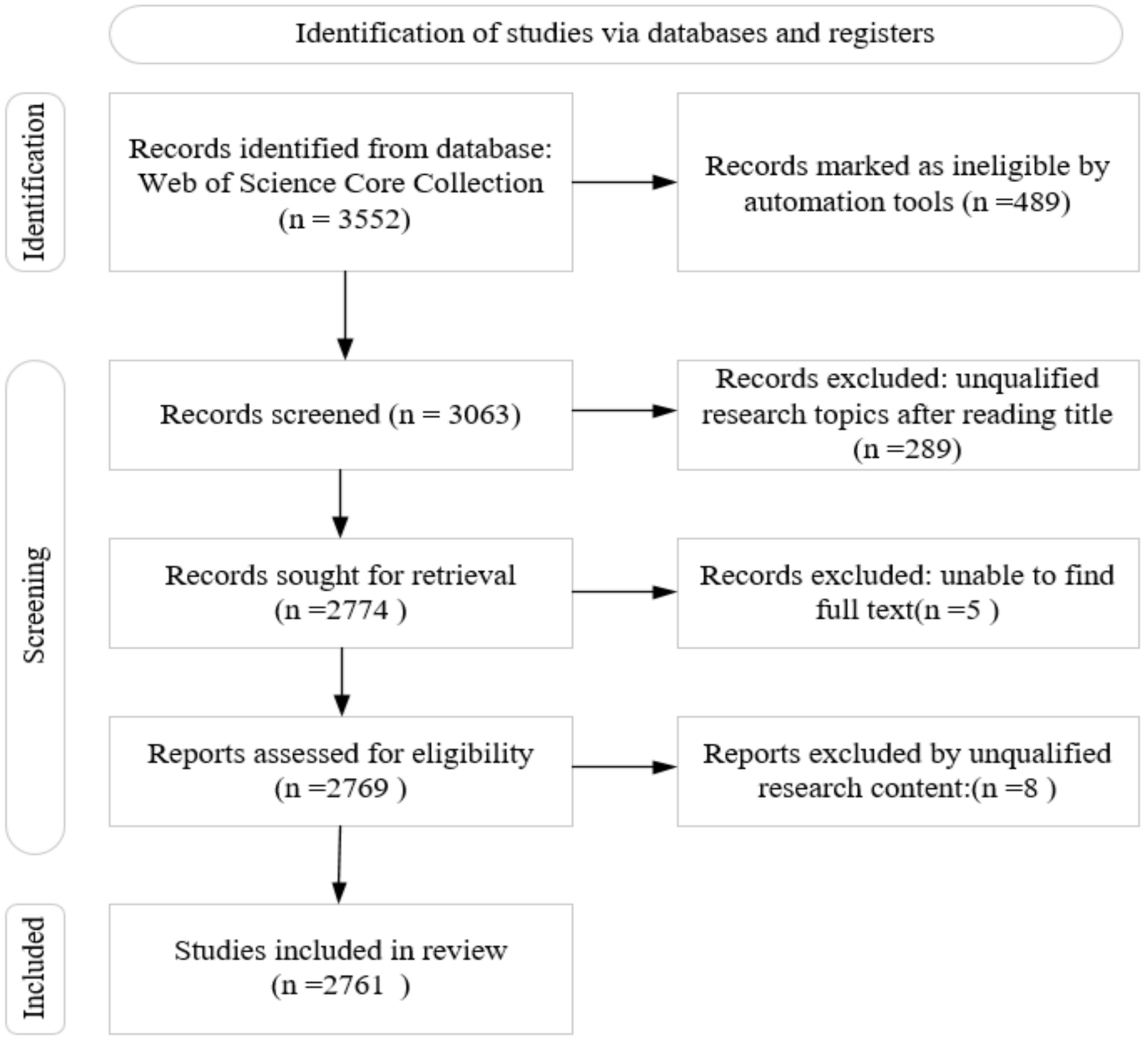
PRISMA flow chart for the search process.

Regarding the bibliometric analysis of OHISB, this study uses a visual scientific knowledge graph based on CiteSpace to explore the frontier of subject development in this field. CiteSpace is a widely recognized scientometric software that sorts complex and diverse relationships, development trends, research hotspots, and frontiers within a certain knowledge field. Accurate and comprehensive data screening is required to explore the subject evolution direction and frontier hot development trends between knowledge units and networks. As the world’s largest comprehensive academic database platform covering most disciplines, the Web of Science contains nearly 9,000 references cited in core academic journals and papers in various disciplines, such as social sciences. This is an ideal way to understand and track the latest progress in related research topics. The search strategy in this study follows PRISMA guidelines. To ensure that the data source has substantial authority, representativeness, and academic influence, data in this study come from the Web of Science Core Collection. First, this study determined the search expression for the topic. Among them, the relevant expressions corresponding to “online” mainly include “Internet” and “Online.” The relevant expressions corresponding to seeking behavior are “seeking behavior” and “seeking behavior.” The English expression “online” was combined with “health information” and “seeking behavior,” respectively. A subject search was performed in the Web of Science Core Collection with the search formula: TS = (Online AND health information AND seeking behavior) or TS = (Internet AND health information AND seeking behavior) or TS = (Online AND health information AND seeking behavior) or TS = (Internet AND health information AND seeking behavior). A set of 3,552 publications were initially retrieved. Then, records were marked as ineligible by automation tools for 489 of them (e.g., keyword matching and duplicate removal). This study also applied field-specific filters (e.g., title, abstract, keywords) to identify the more relevant studies. 2,774 publications were identified as retrieval after screening the titles for relevance. Records were included only if the researchers could find the full text. Therefore, 2,769 publications were assessed for eligibility. Finally, 2,761 publications were included by excluding unqualified research content (see Figure 1). Regarding the issues of data validity and comprehensiveness, CiteSpace is different from other systems in that there is no need to exclude irrelevant literature when collecting data, so this article does not need to exclude sample data that is irrelevant to the topic (8).

## 3 Research status

### 3.1 Overview of the number of publications

**Figure 2:**
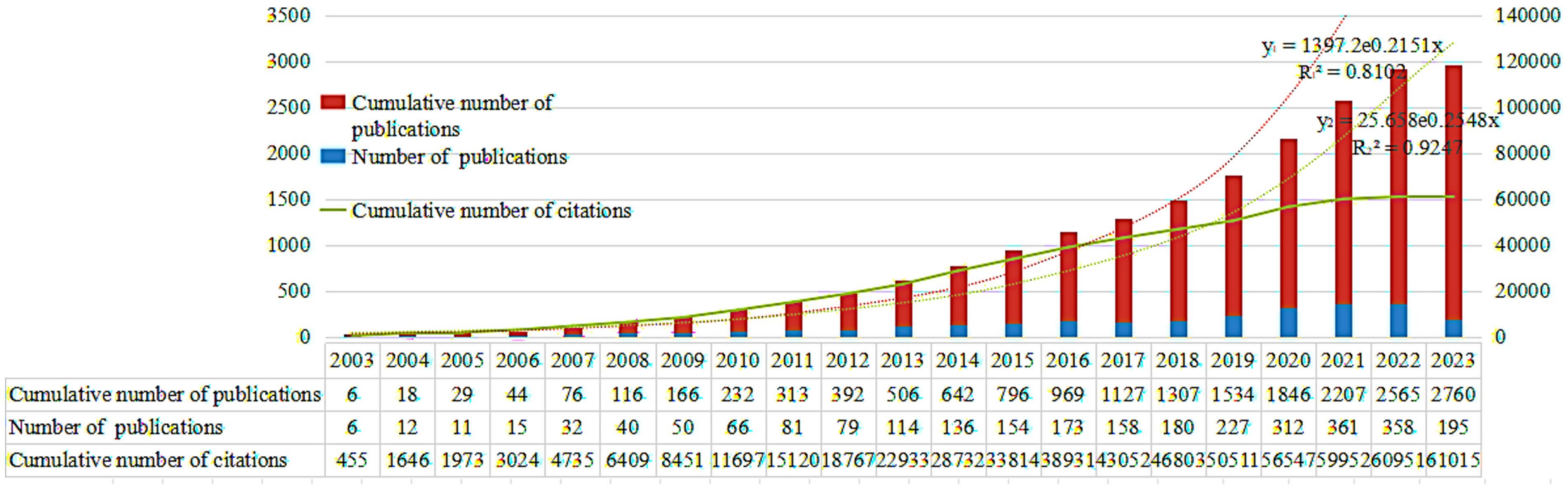
Annual publication volume, citation volume, and publication trend of OHISB.

Figure 2 shows the statistical data of the OHISB study from 2003 to 2023, including the sample literature quantity, cumulative literature quantity, cumulative citation quantity, and their fitting size. Overall, there was a continuous growth trend. The changing trend in the number of studies is an important measurement indicator for judging the development level and technological output of a certain discipline’s research field. As shown in Figure 2, the relevant literature on OHISB first appeared in 2003. According to the time trend of annual publication volume, the research stage can be divided into three stages: budding, growth, and maturity. Specifically, during the budding period from 2003 to 2012, the annual publication volume was less than 100 articles. The growth stage is from 2013 to 2018, with over 100 articles per publication. Since 2019, research on OHISB has been in a mature stage. The number of relevant literature publications exceeded 200 per year, with a significant increase in the annual publication volume. Based on the exponential function of the cumulative number of publications and citations, the fitting results show that R2 is greater than 0.8, indicating a good fitting effect. Moreover, the actual and theoretical values of the cumulative number of publications and citations are increasing each year, indicating that research on OHISB is becoming more mature, and research on OHISB is increasingly receiving academic attention.

### 3.2 Journal and discipline analysis

Academic journals are important carriers for disseminating academic research results through peer reviews. Different academic journals significantly differ in operating models, positioning, and influence. Disciplines reflect scientific research fields with relatively independent knowledge systems. Clarivate’s JCR divides all journals into 21 major categories and 254 sub-categories. Each journal is affiliated with at least one subcategory.

Table 1 lists the top 15 source journals with the most published literature on OHISB and the sub-disciplinary categories to which they belong. This can further understand the relevant research hotspots and the quality of the research results. This article chooses to evaluate the comprehensive influence of the journal based on the impact factors, JCR partitions, citation frequency, H-Index, and other indicators of major journals in 2022, announced on the JCR official website on June 28, 2023. Table 1 shows the distribution of the top 15 source journals and their subject categories for OHISB research. The journals with the largest number of publications are, in order, “J MED INTERNET RES,” “HEALTH COMMUN,” and “INT J ENV RES PUB HE,” “J HEALTH COMMUN,” “PLOS ONE” and “BMC PUBLIC HEALTH,” these six journals have published more than 50 articles, totaling 632 articles, accounting for 22.89% of the total number of articles published, which shows that this journal focuses more on related research in the field of OHISB. Comprehensive analysis of the academic level and influence of journals from indicators such as impact factor, JCR partition, citation frequency, and H-Index, except that the journal “INT J ENV RES PUB HE” is not included in the latest JCR journal citation report, “Online Health Among the top 15 source journals for research related to “Information-seeking Behavior,” 7 journals have an impact factor of more than 4 and are all distributed between Area 1 and Area 2 of the JCR division, and 8 journals have been cited more than 1,000 times. Eight books with indices greater than 20. Among them, “J MED INTERNET RES” has significant advantages compared with other journals regarding the number of publications, impact factor, JCR partition, citation frequency, and H-index. The total number of publications reached 275. The impact factor in 2022 is 7.4 and is in JCR Area 1. It has been cited 11,530 times and has an H-Index of 57. Therefore, it can be judged from the impact factor, JCR partition, citation frequency, and H-Index that the research topic OHISB has been recognized by journals with a comprehensive influence and has produced many high-quality scientific research results.

**Table 1:** The top 15 source journals and their subject categories for OHISB.

Journal title	Number of publications	2022JIF	JCR category	citations	H-Index	JCR subcategories
J MED INTERNET RES	275	7.4	Q1	11,530	57	HCS&S; MI
HEALTH COMMUN	78	3.9	Q1	1,447	24	C; HP&S
INT J ENV RES PUB HE	78	None	None	1,137	17	None
J HEALTH COMMUN	76	4.4	Q1; Q2	2082	27.4	C; IS&LS
PLOS ONE	67	3.7	Q2	1729	25.85	MS
BMC PUBLIC HEALTH	58	4.5	Q2	1,067	18.4	P, E&OH
HEALTH INFO LIBR J	49	3.8	Q2	530	10.82	IS&LS
INFORM RES	42	0.8	Q4	327	7.79	IS&LS
INT J MED INFORM	35	4.9	Q1; Q2	1,340	38.29	HCS&S; CS&IS; MI
COMPUT HUM BEHAV	34	9.9	Q1	1,338	39.47	P&E; P&M
FRONT PUBLIC HEALTH	31	5.2	Q1	112	6	P, E&OH
PATIENT EDUC COUNS	30	3.5	Q1; Q2	961	32.1	SSI; P, E&OH
FRONT PSYCHOL	28	3.8	Q1	282	7	P&M
BMJ OPEN	23	2.9	Q2	190	8.3	MG&I
JMIR PUBLIC HLTH SUR	23	8.5	Q1	633	27.57	PE&OH

HCS&S, HEALTH CARE SCIENCES & SERVICES; MI, MEDICAL INFORMATICS; C, COMMUNICATION; HP&S, HEALTH POLICY & SERVICES; IS&LS, INFORMATION SCIENCE & LIBRARY SCIENCE; MS, MULTIDISCIPLINARY SCIENCES; P, E&OH, PUBLIC, ENVIRONMENTAL & OCCUPATIONAL HEALTH; CS&IS, COMPUTER SCIENCE, INFORMATION SYSTEMS; P&E, PSYCHOLOGY, EXPERIMENTAL; SSI, SOCIAL SCIENCES, INTERDISCIPLINARY; MG&I, MEDICINE, GENERAL & INTERNAL; P&M, PSYCHOLOGY, MULTIDISCIPLINARY.

**Figure 3:**
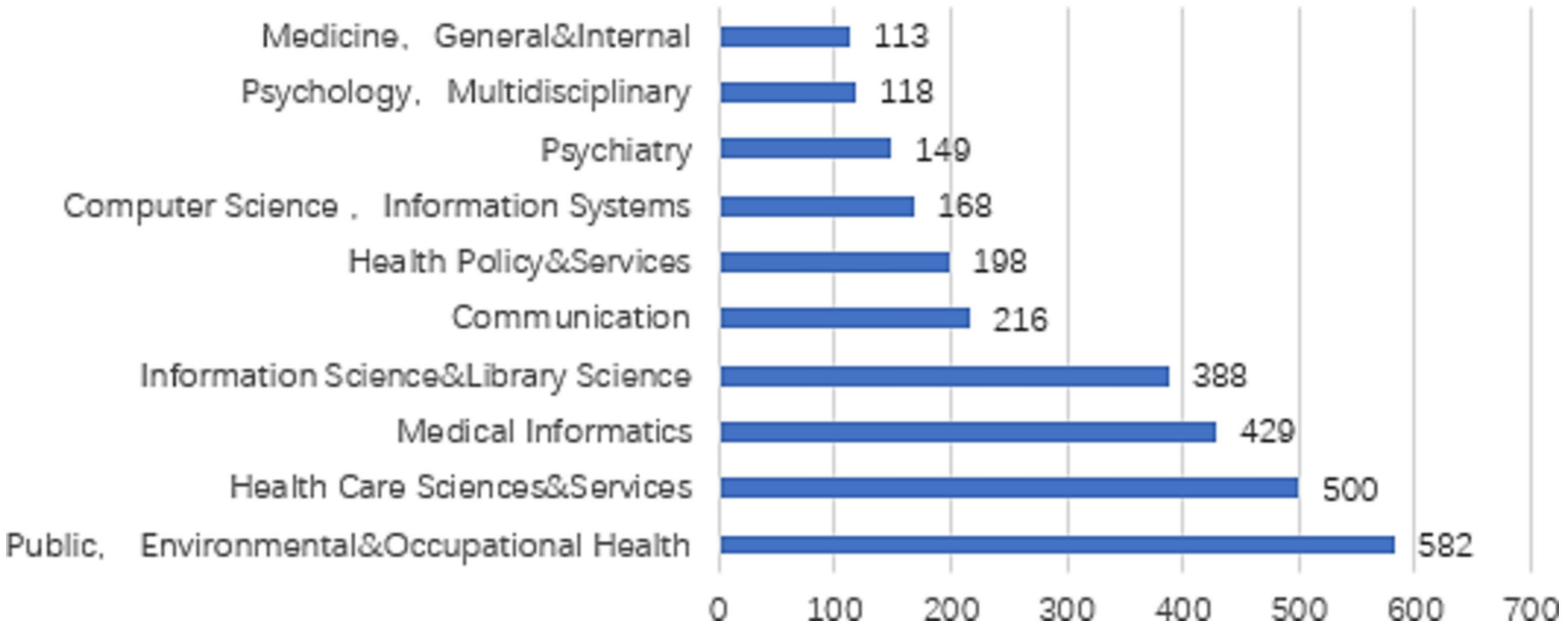
Discipline distribution of research on OHISB.

In terms of subject categories (see Figure 3), JCR sub-categories are on the y-axis, and the number of publications in the x-axis. The statistical results of the sample data obtained in this study show that the research on OHISB involves 135 JCR sub-categories, including public utilities, environment, and occupational health (Public, Environmental & Occupational Health) 582 articles, Health Care Sciences, and Services (HEALTH CARE SCIENCES & SERVICES) 500 articles, Medical Informatics (MEDICAL INFORMATICS) 429 articles, Information Science and Library Science (INFORMATION SCIENCE & LIBRARY SCIENCE) 388 articles, Communication (COMMUNICATION) 216 articles, which shows that multidisciplinary research oriented toward OHISB has been formed.

### 3.3 Cooperation network analysis

Based on the different granularities of cooperation entities, cooperation network analysis can be divided into macro-level national (regional) cooperation, micro-level institutional cooperation, and micro-level author cooperation analyses.

Developing a country’s macro-social environment has an important impact on promoting a certain research field. Therefore, analyzing the spatial characteristics of countries (regions) in the research field of OHISB will help to identify the country’s performance in this field. Research Strength and Scientific Research Cooperation. This study conducted a network analysis of cooperation between countries on 2,761 documents and counted the number of publications and centrality, as shown in Table 2. Based on statistical data, it was found that 98 countries (regions) worldwide conduct research on OHISB. According to the statistical results of the number of publications and centrality of relevant countries (regions) in the research field of OHISB in Table 2, the United States ranks first in terms of the number of publications and centrality, followed by China and Australia., United Kingdom, Canada, Germany, South Korea, Italy, Netherlands, and Singapore. Generally, the main research countries (regions) on OHISB are mostly distributed in North America, Europe, China, and other Asian countries. The United States is in the leading position in the number of publications and centrality in the research field of OHISB. Since 1980, the United States has proposed and implemented health promotion plans every 10 years. They are “Healthy Citizenship 1990: Promoting Health and Preventing Disease,” “Healthy Citizenship 2000: Promoting Health and Preventing Disease,” “Healthy Citizenship 2010: Understanding and Improving Health,” and “Healthy Citizenship 2020: Achieving Goals for Measuring Progress and Eliminating Health” Gap,” which is of great significance to improving national health and achieving sustainable development (9). Australia proposed a universal medical security system in the 1980s, and the United Kingdom and Canada launched their first health strategies based on their own national health services and medical security systems in 1989 and 2001, respectively. With the development of the social economy, China officially issued the “Healthy China 2030” Planning Outline in October 2016, marking that the country has elevated people’s health to a national strategic level and placed it in a priority development position. Therefore, national policies affect the number of publications on OHISB. In terms of centrality, the size of the centrality value represents the intensity of cooperation between countries. China is only 0.07, much lower than the United States, Australia, the United Kingdom, and Italy.

**Table 2:** Top 10 countries (regions) with the most publications in the research field of OHISB.

S. No	Country	Number of articles published (articles)	Centrality	Proportion (%)
1	USA	1,171	0.44	42.41
2	People R China	336	0.07	12.17
3	Australia	284	0.17	10.29
4	England	245	0.22	8.87
5	Canada	157	0.07	5.69
6	Germany	136	0.07	4.93
7	South Korea	93	0.02	3.37
8	Italy	92	0.08	3.33
9	Netherlands	80	0.06	2.90
10	Singapore	69	0.01	2.50

The results in Table 2 highlight the dominant role of the United States and China in OHISB research, with the United States contributing 42.41% of articles and China contributing 12.17%. There are several reasons why these countries dominate OHISB publications, including their large populations, advanced research infrastructure, and the growing relevance of digital health tools. Cultural differences between the United States and China may influence OHISB. In the United States, individualism and personal empowerment often drive people to actively seek out health information online, especially when faced with high medical costs or insurance complications. In contrast, China, which has a collectivist culture, is likely to see more people turn to online healthcare resources as a way to address healthcare disparities in society however, it is also influenced by the government’s increasing focus on digital healthcare initiatives. These trends underscore the importance of understanding the changing dynamics of health information-seeking behavior in response to the healthcare system, economic factors, and cultural influences. This illustrates the transnational cooperation between international scholars in the field of OHISB. Therefore, enhancing one’s academic influence and recognition by strengthening external academic exchanges is a key direction that needs further improvement. It is also suggested that other countries strengthen international cooperation, promote joint research, and increase participation in global academic exchanges to improve the country’s contribution and academic recognition in this field.

In addition, scientific research institutions are important organizational forms that lead to academic development and expand the production and dissemination of scientific knowledge. Analyzing from the perspective of institutions, the top 10 institutions with the highest number of publications in the research field of OHISB were obtained according to the ranking rules of decreasing the number of publications, as shown in Table 3. As can be seen from Table 3, scientific research institutions that publish papers are mainly concentrated in American universities, occupying six of the top 10 publications. The discipline with the most collaborative institutions in the OHISB study is centered on the University of California system, which has the highest centrality score (0.14) among the top institutions. This demonstrates its key role in facilitating collaboration across the research network. Other highly collaborative institutions include Harvard University (0.12), the University of Sydney, and the University of North Carolina (both 0.10), highlighting the prominence of US and Australian institutions in driving partnerships. The data shows that US institutions dominate collaboration in this area, while Australian institutions such as the University of Sydney and the University of Melbourne are actively contributing through regional and international partnerships. This interconnectedness strengthens the global research network and facilitates the dissemination of OHISB’s main research findings.

**Table 3:** Top 10 institutions with the most publications in the research field of OHISB.

S. No	Institution name	Country	No of publications	Citation	All cited frequency	H-Index	Centrality
1	University of California System	USA	79	1851	23.43	25	0.14
2	University of London	England	75	118	17.57	22	0.08
3	State University System of Florida	USA	72	1990	27.64	23	0.09
4	University System of Ohio	USA	68	1,519	22.34	24	0.04
5	Harvard University	USA	64	2,454	38.34	28	0.12
6	University of Sydney	Australia	55	1,329	24.16	18	0.1
7	University of North Carolina	USA	53	1,515	28.58	19	0.1
8	University of Texas System	USA	52	1,408	25.6	19	0.09
9	University of Melbourne	Australia	44	741	16.84	14	0.01
10	University of Toronto	Canada	41	908	21.26	18	0.08

Table 4 lists the top 10 authors’ main research areas in OHISB. Jiang Shaohai is the author with the most publications, with 12 articles published during the sample search period. The author mainly studied OHISB from the perspective of the digital divide (10), influencing factors (11), and social media (12). Zhang Runtong and Lu Xinyi have the same number of publications. Zhang Runtong and Lu Xinyi are both from the School of Economics and Management of Beijing Jiaotong University and have a relatively close cooperative relationship. Based on the summary of papers published by the two scholars, it is found that they mainly focus on the research on factors affecting user compliance in the process of online health information seeking (7), the impact on user trust (13), and research on health access channels (14). Baumann Eva and Link Elena are from the University of Hannover (Universität Hannover) in Germany. The research fields of these two scholars are very similar. They mainly studied online health information seeking from two aspects: digital health participation (15) and influencing factors (16). Dadaczynski Kevin and Okann Orkan, two scholars from different universities in Germany, mainly conduct research on OHISB around e-health literacy. These two scholars were selected as ESI Highly Cited Papers to study the relationship between digital health literacy and online information-seeking behavior among German college students during the COVID-19 pandemic (17). The research fields of other authors with many publications mainly explored health information acquisition channels, health information quality, and health data mining.

**Table 4:** Top 10 authors in the research field of OHISB with a large number of publications.

S. R	Authors name	Publications	Main research areas	Proportion (%)
1	Jiang Shaohai	12	Digital divide, influencing factors, social media, user behavior intentions.	0.45
2	Zhang Runtong	11	User compliance, trust, and channels for obtaining health information.	0.41
3	Lu Xinyi	11	User compliance, trust	0.31
4	Baumann Eva	9	Digital health participation and influencing factors.	0.34
5	Brigo Francesco	8	Health information acquisition channels, data mining	0.30
6	Hornik Robert C	8	Cross-source health information acquisition	0.30
7	Link Elena	8	Digital health participation, user behavior, information avoidance	0.30
8	Dadaczynski Kevin	8	digital health literacy	0.30
9	Okann Orkan	7	digital health literacy	0.26
10	Cruvinel Thiago	7	Health information quality assessment, health data mining and analysis, health literacy	0.26

This table presents a collection of highly cited key studies in OHISB research. The studies covered a wide range of topics, including the use of digital tools such as Google Trends for health research, the impact of social media and peer support on mental health care, and the impact of the COVID-19 pandemic on stress levels and adherence to public health guidelines. In addition, the table highlights investigations into e-health literacy, the quality of health information on platforms such as YouTube, and the efficacy of mobile apps in promoting positive health behavior change. In addition, it highlights the role of online communities in shaping health perceptions and behaviors while examining the characteristics and motivations of individuals seeking health information on the Internet, particularly during major life events such as pregnancy. These studies contribute to the understanding of how individuals navigate and utilize health-related online platforms, revealing the opportunities and challenges that the digital age presents in healthcare communication and decision-making (Table 5).

**Table 5:** Top 10 highly cited studies.

S. R.	Title	Year	Journal	Citation
1	The Use of Google Trends in Health Care Research: A Systematic Review	2014	PLOS ONE	573
2	The future of mental health care: peer-to-peer support and social media	2016	EPIDEMIOLOGY AND PSYCHIATRIC SCIENCES	454
3	Americans’ COVID-19 Stress, Coping, and Adherence to CDC Guidelines	2020	JOURNAL OF GENERAL INTERNAL MEDICINE	451
4	eHealth Literacy: Extending the Digital Divide to the Realm of Health Information	2012	JOURNAL OF GENERAL INTERNAL MEDICINE	411
5	Physician gender and patient-centered communication: A critical review of empirical research	2004	ANNUAL REVIEW OF PUBLIC HEALTH	387
6	eHealth Literacy and Web 2.0 Health Information-seeking Behaviors Among Baby Boomers and Older Adults	2015	JOURNAL OF MEDICAL INTERNET RESEARCH	381
7	Sharing Health Data for Better Outcomes on PatientsLikeMe	2010	JOURNAL OF MEDICAL INTERNET RESEARCH	378
8	Using the Internet for Health-Related Activities: Findings from a National Probability Sample	2009	JOURNAL OF MEDICAL INTERNET RESEARCH	358
9	YouTube for Information on Rheumatoid Arthritis - A Wakeup Call?	2012	JOURNAL OF RHEUMATOLOGY	348
10	Can Mobile Phone Apps Influence People’s Health Behavior Change? An Evidence Review	2016	JOURNAL OF MEDICAL INTERNET RESEARCH	346

## 4 Research hotspots and development trends

### 4.1 Research hotspot analysis

#### 4.1.1 High-frequency keyword statistical analysis

Keywords can provide a concise summary of the main research content of the literature, and statistical analysis of keywords can help identify research hotspots in this field. This article uses CiteSpace software to obtain the top 20 high-frequency keywords in the research field of OHISB, as shown in Table 6. Excluding self-directed keywords that are consistent with the topic search expression, the top five most frequently occurring keywords are “Care,” “Impact,” “Communication,” “Social media,” and “Knowledge.” The top five keywords ranked according to centrality are “Care,” “Knowledge,” “Communication,” “Risk,” “Impact,” “Quality” and “Social media.” Therefore, from the perspective of frequency and centrality, care is the main purpose of OHISB, and social media is the main channel for obtaining online health information. In addition, online health information is regarded as a type of knowledge by users, and knowledge sharing is achieved through communication (18).

**Table 6:** OHISB research keyword frequency and centrality.

S. R	Keywords	Frequency	Centrality
1	Internet	690	0.10
2	Behavior	452	0.09
3	Health information	359	0.07
4	Information	279	0.04
5	Seeking	275	0.04
6	Health	263	0.05
7	Care	256	0.10
8	Impact	254	0.04
9	Communication	249	0.06
10	Information-seeking	240	0.04
11	Social media	230	0.03
12	Knowledge	200	0.08
13	Online	190	0.02
14	Internet use	178	0.04
15	Quality	166	0.04
16	Behaviors	160	0.06
17	Risk	159	0.06
18	Web	145	0.03
19	Attitude	140	0.07
20	Health literacy	130	0.01

#### 4.1.2 Keyword cluster analysis

**Figure 4:**
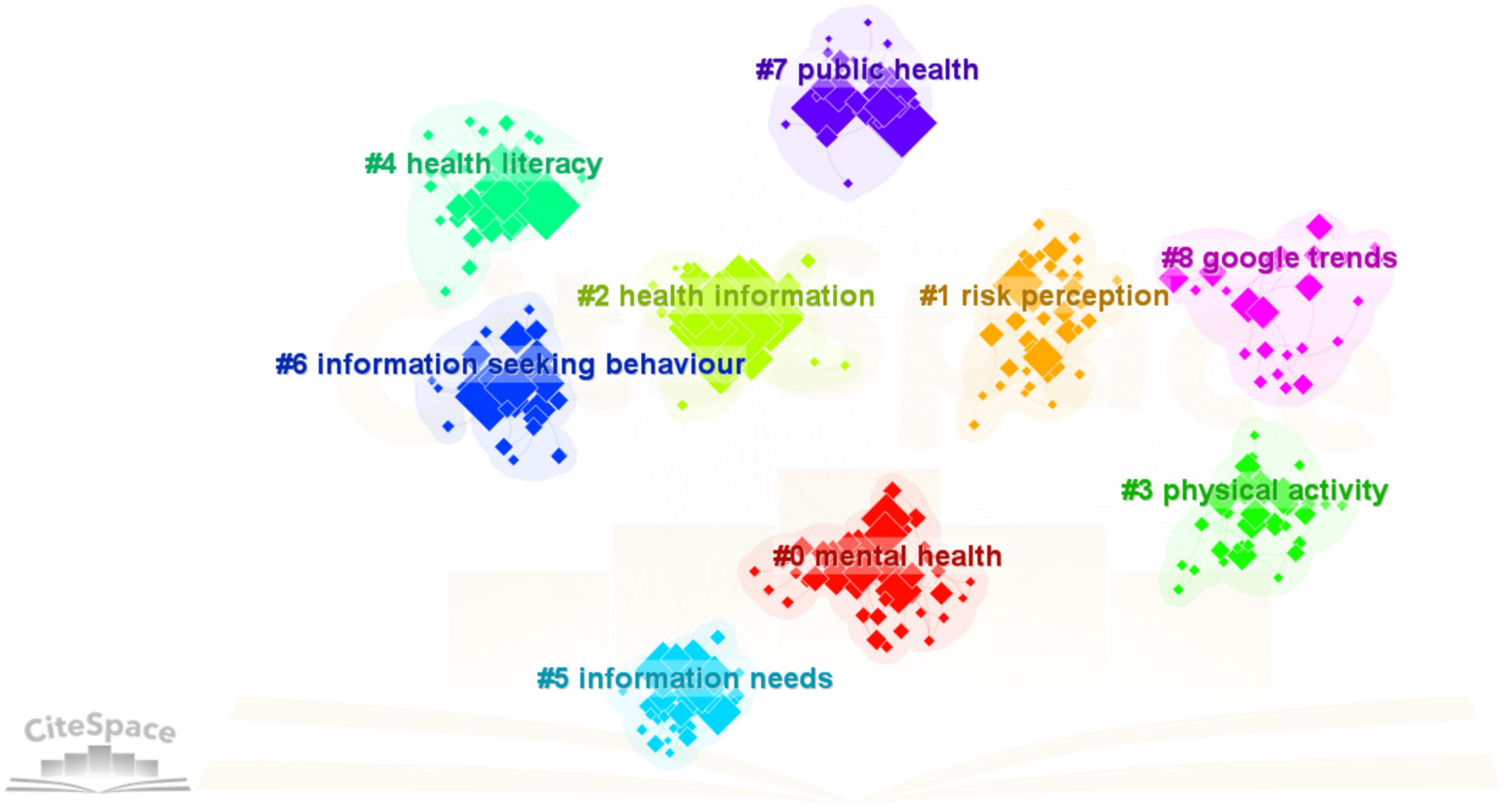
Keyword clustering in the study of OHISB.

This article uses CiteSpace software to conduct keyword cluster analysis on sample documents. It obtains a topic cluster visualization map (see Figure 4) in the research field of OHISB. The representative keywords of each cluster were juxtaposed (see Table 7). This clustering structure’s module value (Modularity Q) was 0.4751 and greater than 0.3 and the Mean Silhouette value (mean silhouette) was 0.7377 and greater than 0.5. Therefore this clustering structure is considered to have good stability and clarity. In addition each cluster’s silhouette value (silhouette) is greater than 0.6 indicating that the members of each cluster are highly similar. This topic clustering map involves nine major clusters: #0 Mental health, #1 Health information, #2 Risk perception, #3 Physical activity, #4 Health literacy, #5 Information needs, #6 Information-seeking behavior, #7 Public health and #8 Google trends. Based on further sorting and summarizing the clustering tags the hot topics in the study of OHISB are summarized into the following six categories:

**Table 7:** Representative keywords of topic clustering in OHISB.

Cluster number	Scope	Outline value	Average year	Representative keywords
#0	42	0.778	2015	Mental health; Depression; University students; Help-seeking; Anxiety
#1	39	0.79	2016	Risk; Perception; Risk; Communication; Perceived risk; Covid-19; Exposure
#2	37	0.841	2009	Health information; Health information-seeking; Information-seeking; social media
#3	34	0.686	2014	Physical activity; Health promotion; Health behavior; College students; Exercise
#4	33	0.682	2013	Health literacy; E-health literacy; Online health information-seeking; Qualitative research; Perception
#5	29	0.749	2009	Information needs; Information-seeking behavior; Needs; Decision making; Breast cancer
#6	29	0.693	2012	Information-seeking behavior; Consumer health information; Health information needs; Online health information
#7	28	0.769	2008	Public health; Patient education; Men
#8	26	0.755	2016	Google trends; Cyberchondria; Health anxiety; Big data; Patient compliance

Cluster 1: Mental health research. Cluster topic #0 (mental health) is the largest group in foreign OHISB research. This cluster label includes mental health, depression, and university students, and help-seeking and Anxiety are representative keywords. Current highly cited literature and hot articles on mental health mainly focus on mental health care methods (19) and factors affecting mental health (20), among which college students are the key targets of mental health research (21). The survey results of the “2022 Global Risks Report” released by the World Economic Forum show that mental health has become one of the top 10 risks in the world. The World Health Organization (WHO) also calls on all countries to ensure everyone receives mental health support. In addition, affected by internal and external factors such as academic pressure, interpersonal relationships, employment planning, living habits, family, and social support, the mental health problems of college students have become an important issue of national health concern in many countries (22).

Cluster 2: Social media. This clustering is mainly obtained based on cluster topic #2 (health information), cluster topic #5 (information needs), and cluster topic #6 (information-seeking behavior), which mainly involve online health information, health keywords such as information-seeking (health information-seeking), decision making (decision making), and social media (social media). Health information-seeking on social media provides decision-making support for health information and satisfies consumers’ social and emotional needs (23). However, the quality and authority of health information on social media are increasingly challenged, affecting consumer participation behavior. Therefore, improving and standardizing the social media health information ecosystem is currently a research hotspot (24).

Cluster 3: Research on user health literacy. This cluster mainly involves theme #4 (health literacy), including health literacy, e-health literacy, online health information-seeking, qualitative research, cognition (perception), and other keywords. A user’s ability to find, evaluate, and use online health information is affected by their health literacy or e-health literacy levels. Users with higher health literacy can formulate online search strategies more effectively and obtain higher-quality health information sources (25). Mobile apps play a huge positive role in developing e-health and m-health services for people with low health literacy (26).

Cluster 4: Public Health Management Research. Cluster theme #1 (risk perception), cluster theme #3 (sports activities), and cluster theme #7 (public health) can be summarized as public health management research, mainly including risk perception (risk perception), risk communication (risk communication), new coronavirus (Covid-19), physical activity (physical activity), health promotion (health promotion), public health (public health), and patient education (patient education). Public health management ensures public health, social development, and national stability. The public-health-themed papers in this study mainly explore how the public with different health literacy levels search and share health information through different forms of Internet media when facing a public health crisis (27) and identify incorrect health information on the Internet (24).

Cluster 5: Google. This cluster is mainly centered on cluster theme #8 (Google Trends), which includes Google Trends, Cyberchondria, Health anxiety, big data, patient compliance (patient compliance), and other keywords. Google search engines account for nearly 90% of the US market. The Google search engine based on big data will help to understand better, monitor, and predict users’ various health information search patterns, such as influenza, mental health etc., temporal patterns, or geographical differences (28). Although using internet tools such as Google to search for online health information has advantages in terms of convenience, cost, and time, excessive online health-seeking behavior is also accompanied by the amplification of health anxiety and the emergence of cyberchondriacs. Cyberchondriacs reduce patients’ trust in doctors, further increase the tendency of self-medication, and reduce treatment compliance, which can easily lead to health risks (29).

The analysis reveals important links between the clusters identified in the OHISB study, underscoring the interdisciplinary nature of the field. Cluster #0 (mental health) and Cluster #2 (social media) are closely linked, as social media platforms are an important tool for raising mental health awareness and seeking help, especially among college students. In addition, Cluster #2 (social media) and Cluster #3 (Health literacy) intersect, highlighting the impact of health literacy on users’ ability to evaluate and navigate health information shared on social media. Cluster #4 (Public Health Management Research) is linked to Cluster #2 through the key role of social media in risk communication and public health education during crises such as COVID-19. In addition, cluster #3 (health literacy) directly affects public health outcomes (cluster #4) because individuals with higher health literacy are better able to understand and respond to public health guidelines. Finally, Cluster #5 (Google Trends) is related to Cluster #4 through the use of search data to monitor public health trends and is related to Cluster #0 through its application in analyzing mental health patterns, including geographic and temporal changes in anxiety and depression-related searches. These interconnections highlight the integration of mental health, social media, health literacy, public health, and digital tools in shaping health information-seeking behaviors and trends.

### 4.2 Development trend analysis

**Figure 5:**
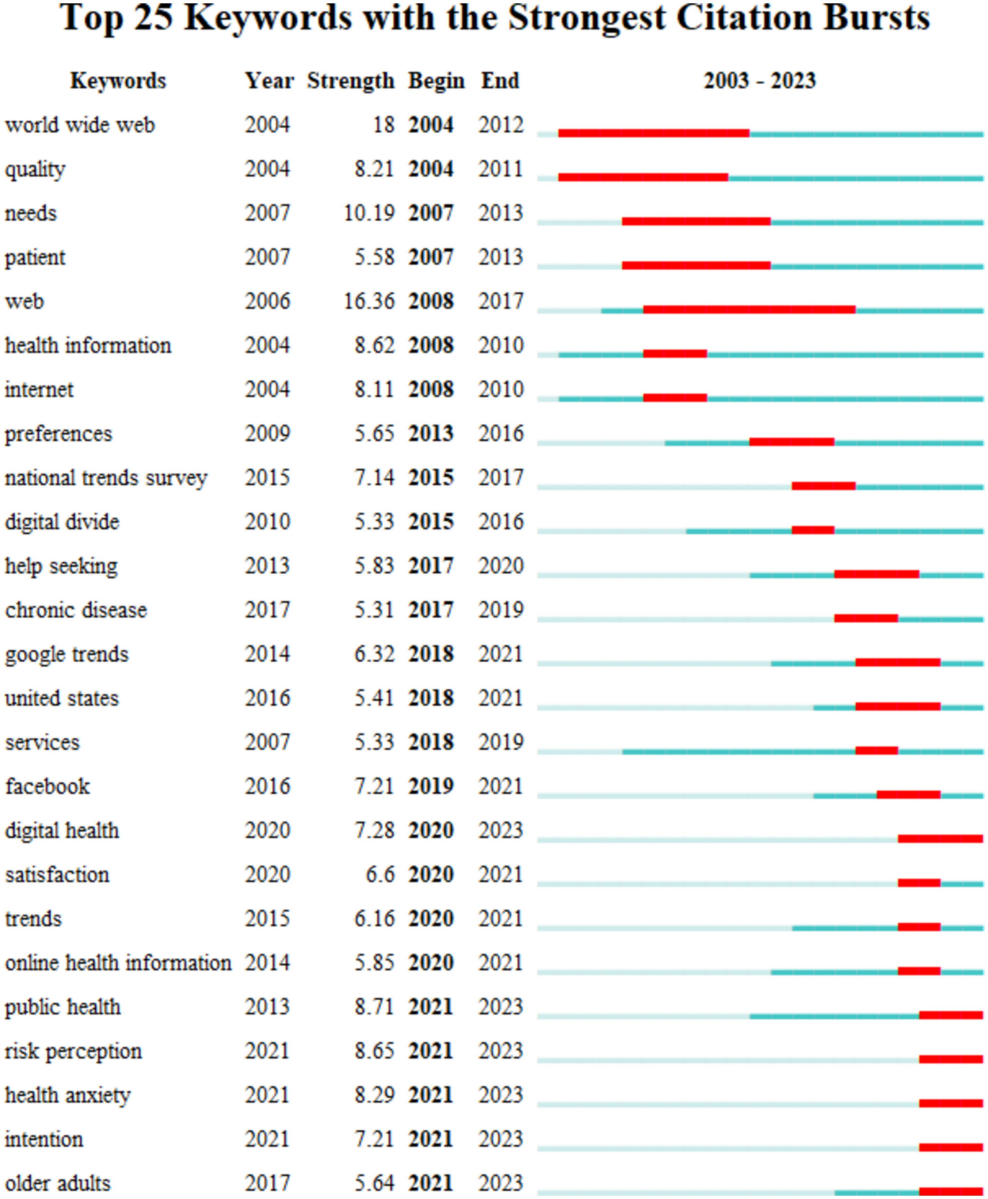
Keyword bursts of OHISB.

Time-series analysis of keywords considering time factors can effectively identify scientific frontiers and emerging trends in a certain research field. Therefore, this study used the CiteSpace software to detect keyword emergence in the relevant literature on OHISB and obtained 25 keywords with the highest emergence intensity. Based on the comprehensive analysis of three aspects: emergence starts time, emergence ends time, and emergence intensity, 7 of the 25 keywords are concentrated in 2004–2010, among which “World Wide Web,” “Web” and “Needs.” The longer prominence intensity and duration indicate that this stage mainly focuses on the impact of Internet technology on health information-seeking behavior. The keyword “Preference” emerged in 2013, and the National Trends Survey of Health Information and Google Trends emerged in 2015 and 2018, respectively. This illustrates the importance of using survey and big data methods to understand, mine, monitor, or predict OHISB and public interests and preferences. In addition, the keyword “Facebook” has the strongest emergence intensity, which shows that social media is the main channel for users to search for online health information at this stage. From 2020, keywords with higher emergence intensity include public health, risk perception, health anxiety, and digital health. In recent years, outbreaks of global public health emergencies have caused countries to pay more attention to the prevention and management of public health. The public has become more sensitive to health risk perceptions, leading to the emergence of psychological problems such as health anxiety. Against this background, using information technologies, such as artificial intelligence and big data, to carry out digital health activities is significant to protect public health and improve national public health levels (Figure 5).

## 5 Conclusion and recommendations

This article uses CiteSpace software to conduct a visual analysis of the status, hot spots, and development trends of OHISB research from 2003 to 2023 and obtains the following research conclusions in response to the research questions:

First, in terms of overall research trends, according to the statistical results of the number of publications and the cumulative number of citations, the number of studies on OHISB has shown a steady growth trend, and the research scope has gradually expanded. This shows that OHISB is a hot issue in current academic research. With the increasing prominence of digital health tools, social media platforms, and online health information sources, these factors directly impact the way individuals’ access, evaluate, and use health-related information. This has practical implications for healthcare providers, policymakers, and public health organizations, who must address issues such as misinformation, health literacy, and the quality of online health resources. Regarding journals and subject distribution, academic achievements related to “online health-information-seeking behavior” are recognized by many high-quality journals and involve multiple interdisciplinary fields. Regarding cooperation networks, the United States is in a leading position in this field. Still, the relevant cooperation networks are mostly based on intra-country and intra-institutional cooperation. The cooperative network structure is relatively simple, and it is necessary to strengthen further and deepen the structure, extent, and number of scientific research cooperation teams.

Second, regarding research hotspots, research on OHISB tends to integrate with the development of information technology. Rapid changes in information technology have led to the continuous enrichment of online health information-seeking channels, such as online search engines, various medical and health websites, mobile apps, Weibo and other social networks, and medical and health databases. In addition, with the application of digital technologies, such as big data, artificial intelligence (AI), and cloud computing, users’ OHISB is transitioning from informatization and digital development to intelligence, thereby realizing intelligent health between humans and machines—information exchange. AI, for example, is going beyond traditional search engines and changing the way people find information. However, the evolution of information-seeking poses challenges, especially for older people. The digital divide, the gap in access to and understanding of technology, disproportionately affects the older population. Many older adults may struggle to adapt to AI-driven platforms, which often require a degree of digital literacy and familiarity with new technologies. For example, voice assistants such as Amazon’s Alexa or Apple’s Siri are widely used to get information, play music, or manage tasks. While these tools are particularly beneficial for older adults with reduced mobility or vision impairment, they still need to understand the technology and comfort of using digital devices. Finally, the rapid growth of information technology has created a digital divide for people with low health literacy levels, especially older people, affecting the quality and efficiency of their health information searches.

Third, regarding research frontiers, the research hotspot of OHISB continues to innovate and change. Early related research on OHISB mainly focused on information ecological factors such as information subjects (such as patients), information technology (such as the World Wide Web), and information (such as information quality). Subsequently, relevant research has gradually focused on the impact of individual online health information-seeking preferences, big data, and social media on users’ OHISB. Public health, risk perception, health anxiety, and the older adult are the research frontiers of OHISB.

This study identified clusters that stood out, including mental health, health information-seeking behaviors, health literacy, social media, and public health management. These findings indicate a growing interest in understanding how individuals, particularly those affected by the digital divide, access health information online.

Future research should integrate cutting-edge information technologies such as big data, AI, and cloud computing with the theme of “network health information-seeking behavior” for more detailed integration. Rapid advances in IT have brought digital inequalities, especially for older people and other communities with low health literacy. Future research should focus on understanding how these differences affect the efficiency and quality of searching for health information. To promote health equity in the digital age, it is necessary to investigate ways to bridge these gaps and enhance access to trusted health information for poor communities. The study of OHISB begins to regard health anxiety as an important research field. The relationship between health outcomes, access to online health information, and health anxiety needs further study. Looking at how people with varying degrees of health anxiety browse the Internet and how this affects their health will help investigate the psychological component of seeking health information. By addressing these prospective research goals, scholars can enhance the understanding of OHISB in the digital age and their impact on health promotion, communication, and decision-making.

Going forward, research should focus on further exploring how AI tools can be optimized to meet the needs of underserved populations, especially the older adult. Research into how AI can be incorporated into public health initiatives to improve health literacy, especially in low-resource Settings, will be valuable. Future research could also assess the effectiveness of digital literacy programs in improving health outcomes for these populations and explore the role of AI in addressing health disparities. In addition, it is important to consider cross-cultural differences in how different populations use AI-based health tools, which may help refine global health improvement strategies.

In addition, this study focuses only on English-language publications, and it may exclude relevant research in other languages. To address these issues, this study recommends that future research expand OHISB research by promoting collaboration with researchers from underrepresented regions and incorporating multilingual analysis to ensure more inclusive insights.

## Data Availability

Publicly available datasets were analyzed in this study. This data can be found form Web of Science core collection at the following link: https://www.webofscience.com/wos/alldb/basic-search.
